# Optical microscopy evaluation of root canal filling removal by beginner operators in posterior teeth

**DOI:** 10.25122/jml-2024-0283

**Published:** 2024-06

**Authors:** Bogdan Dimitriu, Ioana Suciu, Oana Elena Amza, Mihai Ciocârdel, Dana Bodnar, Ana Maria Cristina Țâncu, Mihaela Tanase, Maria Sabina Branescu, Mihaela Chirilă

**Affiliations:** 1 Carol Davila University of Medicine and Pharmacy, Bucharest, Romania; 2 Petroleum-Gas University, Ploiesti, Romania

**Keywords:** endodontic retreatment, remaining root canal filling, microscopy

## Abstract

This study analyzed the effectiveness of root canal filling removal in lower molars performed by beginner operators using optical microscopy. A total of 55 mandibular first and second molars with mesial roots exhibiting an average curvature of 10-20° were selected based on preoperative radiographs. Instrumentation was done with ProTaper Gold (Dentsply Sirona) up to F2 (25/.08), using 2ml of 2.5% NaOCl irrigation solution after each file. Root canal obturation was performed using gutta-percha points with cold lateral condensation and Sealapex (Kerr Dental). Coronal fillings were made with composite resin and stored in distilled water for two years. Removal of the root canal fillings was performed with AF Retreatment Rotary (AFRR) and AF Blue R3 (AFBR3) (Fanta Dental Materials) under reciprocating motion with 2.5% NaOCl irrigation. Cross-sections of the coronal, middle, and apical thirds were analyzed at 40x magnification using a STEINDORFF POL microscope with a digital camera. Image analysis was conducted using Image J software, version 1.54, to determine the efficiency of root canal filling removal by percentage. Statistical analysis via one-way ANOVA revealed significant differences between distal and mesial roots (*P* < 0.05). Specifically, for mesial roots, the removal efficiency was 70.65% in the coronal third, 54.66% in the middle third, and 21.32% in the apical third. Significant difficulties were noted due to fractured files, calcifications, and debris accumulation in the isthmuses. The study concluded that the protocol using Fanta files demonstrated significant differences in removal efficiency correlated with root curvature, compounded by the inexperience of beginner operators. The findings highlight the challenges faced by novice practitioners in achieving effective root canal filling removal.

## INTRODUCTION

Adequate removal of root canal filling material is essential for effective orthograde nonsurgical retreatment, particularly in addressing periapical pathology of previously endodontically treated teeth [1-3]. The complexity of endodontic retreatment increases with the intricacy of the endodontic anatomy, the extent of iatrogenic changes, and the age of the root canal fillings [4-7]. Successful retreatment hinges on the complete removal of the root canal filling material, which is paramount for achieving therapeutic objectives [6,8]. This study evaluated the efficiency of root canal filling removal in an in vitro setting, using extracted molars that were previously endodontically treated in vivo. The focus on molars is due to the inherent challenges posed by the complex anatomy of posterior teeth, which often complicates endodontic retreatment.

A unique aspect of this study is the involvement of beginner operators—endodontic residents who typically start their training on extracted teeth before progressing to in vivo procedures. The rationale for selecting beginner operators lies in the significance of understanding how operator experience influences the efficacy of root canal filling removal. Since novice practitioners are in the early stages of skill acquisition, their performance provides critical insights into the challenges faced during training and the potential need for improved educational protocols.

This investigation aims to highlight the difficulties encountered by beginner operators and to quantify the efficiency of their procedures. By focusing on this demographic, the study seeks to underscore the importance of comprehensive training programs that can better prepare novice practitioners for clinical challenges, ultimately contributing to improved patient outcomes in endodontic retreatment scenarios. The findings have broader implications for endodontic education and the refinement of training methodologies, ensuring that future practitioners are well-equipped to perform effective retreatments.

## MATERIAL AND METHODS

This study involved 55 molars that received endodontic treatment at least two years prior, as verified by individual patient records. These teeth were extracted due to advanced periodontal disease, with informed consent obtained from the patients in endodontic clinics.

### Inclusion and exclusion criteria

Inclusion criteria for the study were molars with documented endodontic treatment completed at least two years before extraction and a history of advanced periodontal disease. Exclusion criteria included teeth with fractures, external resorptions, or previous retreatment attempts to ensure consistent study conditions.

### Retreatment procedure

The initial endodontic treatments of these teeth were performed using cold lateral condensation, warm vertical compaction with incremental backfill, and single cone techniques with sealers AH26 and AH Plus. The retreatment process involved beginner operators, simulating the initial stages of endodontic training. First, the coronal restorations, which were resin composite fillings deemed clinically acceptable, were removed. Root canal orifices were then located. Manual files were used initially to negotiate and open the canal space, followed by rotary files without magnification and without exerting excessive pressure, until the operators considered the removal of gutta-percha and sealer to be complete.

### Cross-sectioning and examination

**Figure 1:**
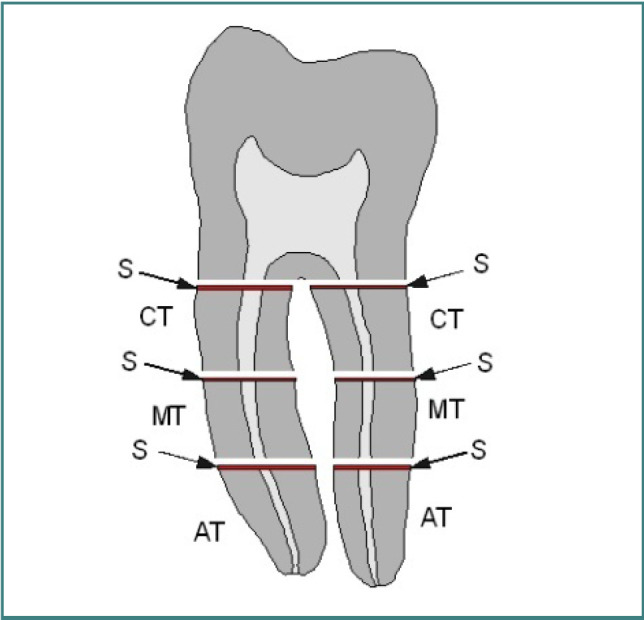
Outline of the molar root cross sections used in this study. Each root was segmented into three sections: coronal (CT), middle (MT), and apical (AT) thirds, with the cross sections (S) examined microscopically.

Post-removal, a Leica Biosystem Microtome RM2125RTS was used to section each tooth's roots into coronal, middle, and apical thirds (Figure 1). Each section was cleaned with an air-water spray to remove cutting debris without dislodging residual root canal-filling materials. Each coronal surface of the root fragments was examined using a Steindorff polarizing microscope at 40x magnification. The rationale for selecting the specific magnification of 40x in this study was based on achieving an optimal balance between detail resolution and field of view. This magnification level allows for a thorough examination of the quality of root canal filling removal and the inspection of root canal dentin. It provides sufficient magnification to detect small residual filling materials, cracks, calcifications, and other anomalies within the root canal while maintaining a broad enough field to view the entire cross-sectional area of the root canal. Photographs were taken of each cross-section, and in cases where two root canals were close together, a single image encompassed both.

### Image analysis

Digital images of the cross-sectional areas of the root canals were analyzed using Image J software, version 1.54. The analysis process involved several detailed steps to ensure accurate quantification of the root canal filling removal. First, each digital image, captured at 40x magnification, was imported into Image J. Software calibration was performed using a known scale embedded in the images to convert pixel dimensions to micrometers (µm). The region of interest (ROI) for each cross-sectional image, representing the root canal area, was manually selected using the ROI tools in Image J. A single ROI encompassing both canals was used to maintain accuracy for images of closely situated root canals. Threshold adjustment was employed to distinguish the remaining root canal filling materials from the surrounding dentin. This involved adjusting the grayscale threshold to highlight the filling material specifically. The area of the highlighted (unremoved) root canal filling material within the ROI was measured in square micrometers (µm^2^), along with the total area of the root canal cross-section. The percentage of the root canal area occupied by the remaining filling material was then calculated by dividing the area of the filling material by the total root canal area and multiplying by 100. The removal efficiency for each section was determined by subtracting this percentage from 100%. These calculated percentages of remaining filling material were recorded for each cross-sectional area, and the data were compiled to identify trends and assess the overall efficiency of the root canal filling removal. To further ensure detailed analysis, five molars presenting the most significant deficiencies in root canal filling removal, including dentinal cracks, calcifications, secondary dentin, and iatrogenic conditions, were selected for focused examination. Data from these samples were entered into tables to integrate the final results and facilitate comparison. This thorough and systematic approach to image analysis and quantification ensured a precise assessment of the endodontic retreatment outcomes.

## RESULTS

**Figure 2:**
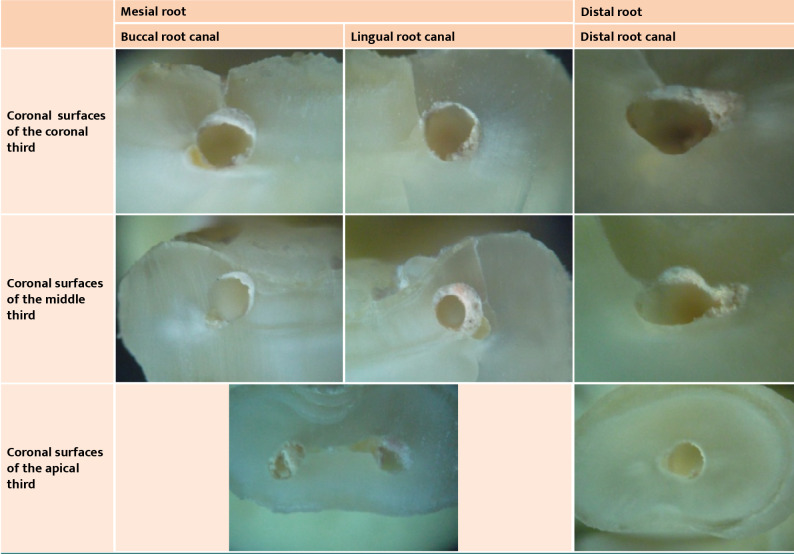
Microscopy images of root cross sections of tooth 47 included in this study (all microscope images used a 40x magnification).

**Figure 3:**
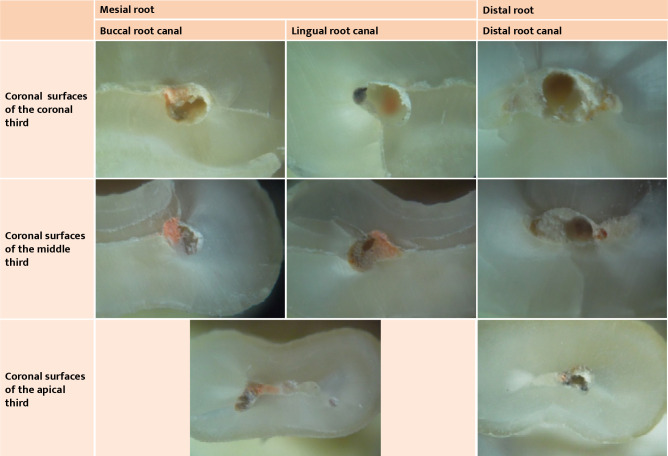
Microscopy images of root cross sections of tooth 46 included in this study (all microscope images used a 40x magnification).

**Figure 4:**
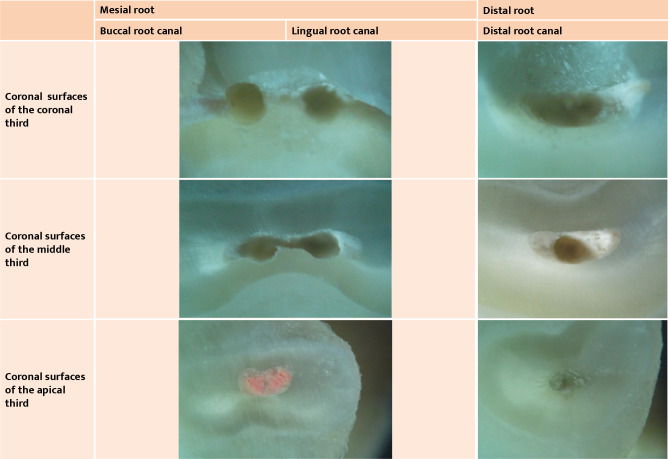
Microscopy images of root cross sections of tooth 36 included in this study (all microscope images used a 40x magnification).

**Figure 5:**
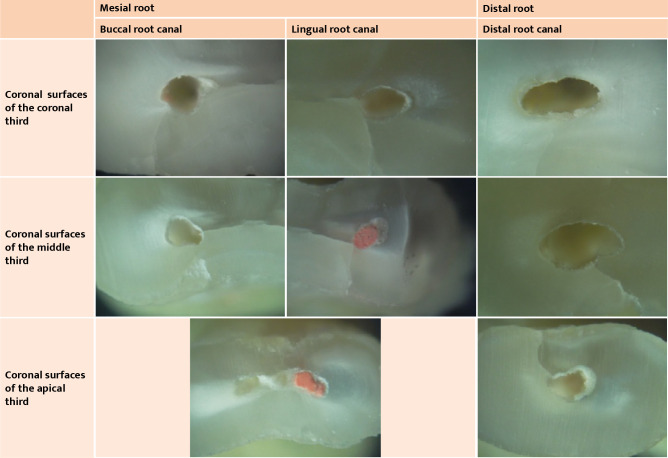
Microscopy images of root cross sections of tooth 47 included in this study (all microscope images used a 40x magnification).

**Figure 6:**
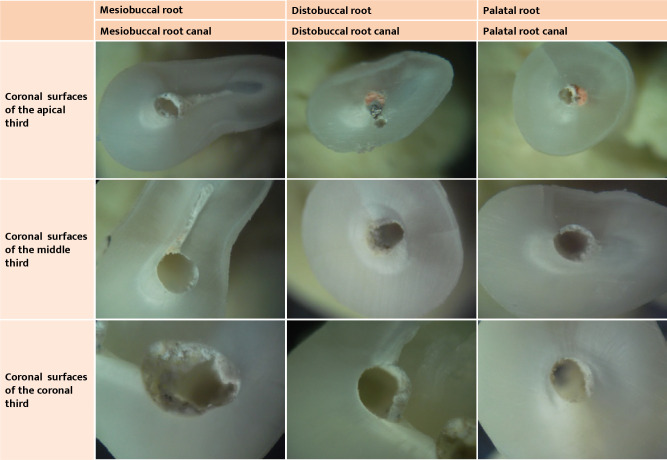
Microscopy images of root cross sections of tooth 27 included in this study (all microscope images used a 40x magnification).

Figures 2 to 6 present the images obtained for the coronal cross-sectional surfaces of each third of the roots of the teeth involved in this study. The digital images were centered on the root canal area. When secondary dentin or calcifications were observed, they were classified as hard tissues rather than materials filling the root canal.

Following these determinations, a number of data were gathered and subsequently centralized in 55 tables, with Tables 1 and 2 selected for exemplification.

**Table 1:** Centralized data for tooth 36 included in this study exemplifying an incomplete desobturation

Tooth 36							
Root	Level	Identified root canals	Percentage of unremoved root canal filling (%); Material type	Percentage of removed root canal filling (%)	Cracks	Calcification/ Secondary dentin	Iatrogenic aspects
Mesial root	Coronal third	Lingual and Buccal isthmus connected	48.93 (Seal+DD)	51.07	-	-	-
	Middle third	Lingual Buccal	46.90 (Seal+DD)	39.64	-	-	-
	Apical third	Lingual and Buccal isthmus connected	100 (Gut+Seal)	0	-	-	-
Distal root	Coronal third	Distal	2 (Seal+DD)	98	-	-	-
	Middle third	Distal	64.39 (Seal+DD)	35.61	1RCC	-	-
	Apical third	Distal	Root canal completely filled with secondary dentin	0	-	SDn	-

**Table 2:** The results obtained after a good quality desobturation

Tooth 36							
Root	Level	Identified root canals	% root canal filling materials unremoved; Material type	Percentage of root canal filling material removal (%)	Cracks	Calcification/ Secondary dentin	Iatrogenic aspects
Mesial root	coronal third	Lingual and Buccal	0	100	-	-	-
	middle third	Lingual and Buccal isthmus connected	9.37 (Seal+DD in the isthmus)	90.63	-	-	-
	apical third	Lingual and Buccal isthmus connected	7.11 (Seal+Gut in the isthmus)	92.89	1NRCC	-	-
Distal root	coronal third	Distal	0	100	-	-	-
	middle third	Distal	0	100	-	-	-
	apical third	Distal	0	100	-	-	-

For a comparative assessment, we present the data concerning the microscopically confirmed acceptable cases of root canal filling materials removal for one case considered significant out of the teeth not illustrated in the previous microscopic images (Table 2).

Considering the average values for the proportion of root canal filling materials removed from each coronal cross-section, we obtained the following results: 70.65% for the coronal cross-section of the coronal third, 54.66% for the coronal cross-section of the middle third and 21.32% for the coronal cross-section of the apical third.

## DISCUSSION

**Figure 7:**
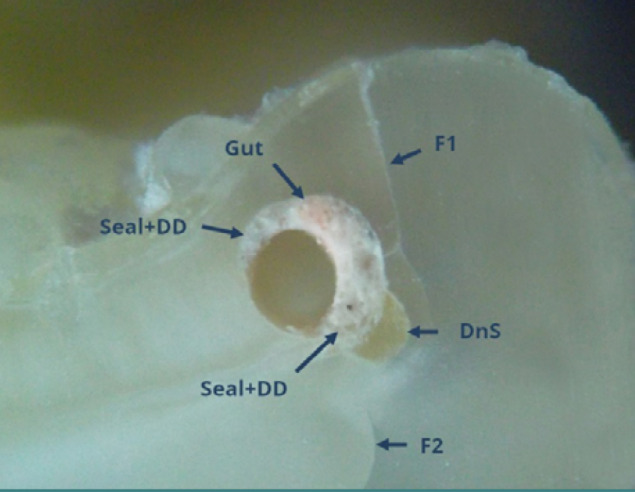
Mesial root of tooth 47, adjacent to the lingual canal: coronal cross-section of the middle third (reflected light microscopy, 40x). Observations include incomplete removal of root canal filling materials, with sealer (Se) containing dentin debris (DD) and gutta-percha (Gut) still present. Two root cracks (C1, C2) are identified: C1 originates from the external root surface, bifurcating near the canal, while C2 follows a curved path without intersecting the canal. Secondary dentin deposition (SDD) is suggested by the non-circular pre-endodontic canal shape.

Secondary dentin deposition was frequently found in root canals, even at the middle third level. It is well known that this could be mistaken by inexperienced evaluators as an older sealer, as noted in other studies [9-13]. Improper access opening can restrict the action of endodontic files to certain parts of the canal [10,14]. This was often due to incomplete removal of the overlying composite restoration (Figure 7).

**Figure 8:**
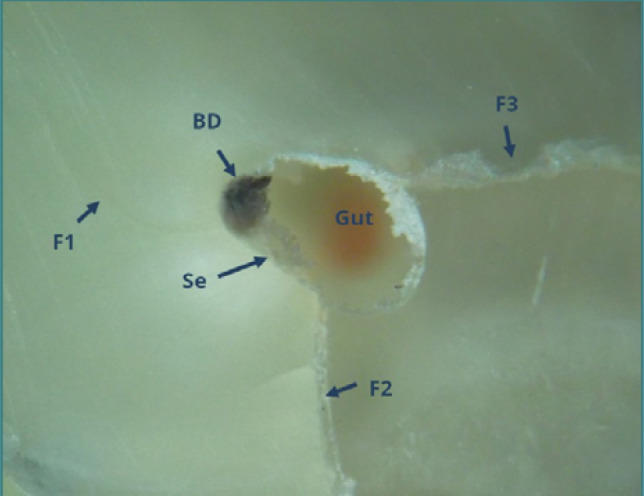
Mesial root of tooth 46, adjacent to the lingual canal: coronal cross-section of the coronal third (reflected light microscopy, 40x). Black debris (BD) of unspecified origin, sealer residues (Se) adhering to the canal walls, and three radial cracks (C1, C2, C3) are noted. C2 is identified as an 'open crack'. Residual gutta-percha (Gut) is visible in the canal's depth, indicated by its orange color.

The presence of radial cracks suggests the untimely use of endodontic files, most likely during the initial shaping of the root canal rather than during the removal of root canal filling materials. This is in line with the literature [15-18] and is supported by the fact that the residents involved in this study were specifically trained not to exert excessive pressure on the root canal walls during instrumentation (Figure 8).

**Figure 9:**
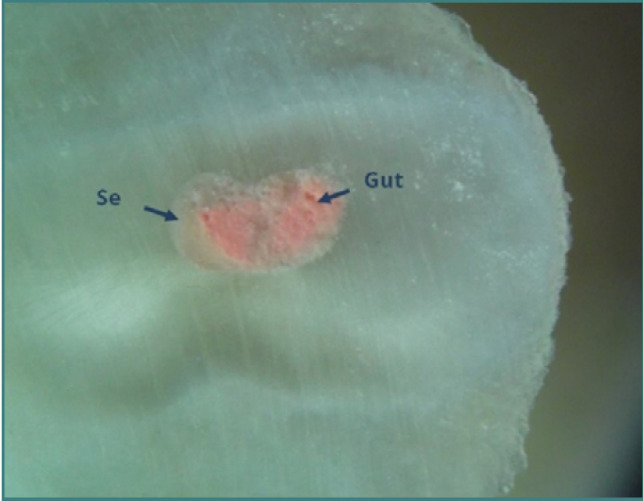
Mesial root of tooth 36: coronal cross-section of the apical third (reflected light microscopy, 40x). The buccal and lingual canals merge into a single canal (Weine type II) at this level. Despite in vitro conditions, complete removal of root canal fillings was frequently unsuccessful in the apical third. A high proportion of sealer (Se) relative to gutta-percha (Gut) was observed. No gutta-percha condensation was performed at this level.

**Figure 10:**
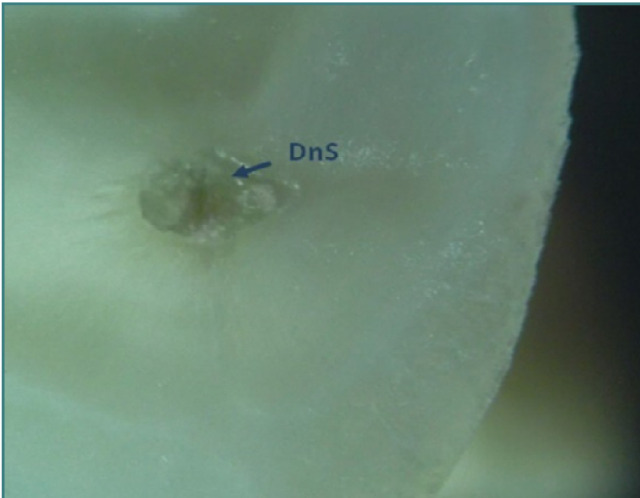
Distal root of tooth 36: coronal cross-section of the apical third (reflected light microscopy, 40x). The root canal is predominantly filled with secondary dentin deposition (SDD), indicating a naturally 'calcified root canal'.

Consistent with published data [19-22], our study found that removing root canal fillings is usually the least successful in the apical third. This can be attributed to the complexity of the endodontic system (Figure 9 – Weine II) or iatrogenic issues such as separated files (Figure 10) [23,24]. Persistent attempts to remove root canal fillings in cases of root canal obliteration by secondary dentin deposition often led to root dentinal cracking or perforations [25-27]. Figure 10 illustrates such a situation in the distal canal of tooth 36, which was completely blocked by secondary dentin deposition.

**Figure 11:**
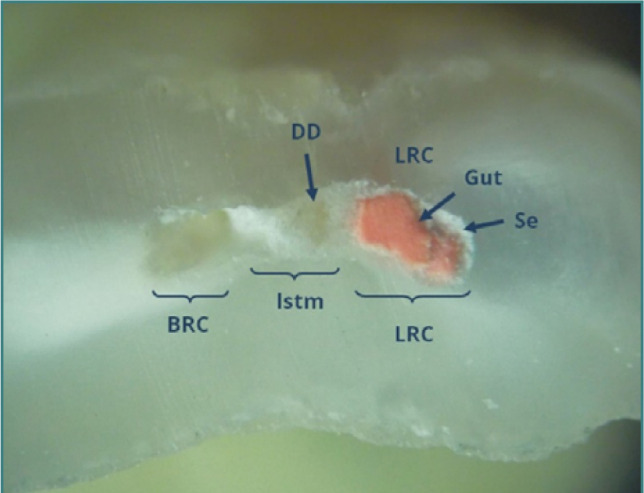
Mesial root of tooth 47: coronal cross-section of the apical third (reflected light microscopy, 40x). An isthmus (Istm) is observed between the buccal root canal (BRC) and the lingual root canal (LRC). The BRC failed to be obturated, while the LRC contains two gutta-percha points (Gut) and sealer (Seal). The isthmus is partially filled with dentinal detritus (DD) and sealer.

**Figure 12:**
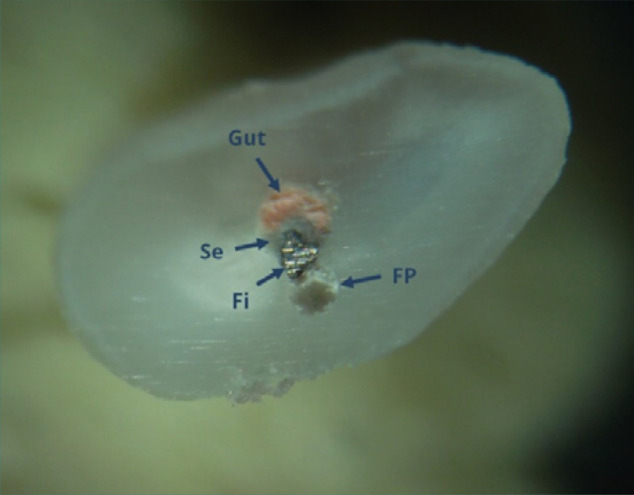
Distobuccal root of tooth 27: coronal cross section of the apical third (reflected light microscopy, 40x). Unremoved root canal filling materials, including laterally condensed gutta-percha (Gut), endodontic sealer (Se), and a separated file (Fi), are present. A false canal (FC) created during the attempt to remove the filling is partially filled with dentin debris.

Isthmuses, endodontic spaces connecting two root canals within the same root, present significant challenges for filling and removing root canal materials [28-30]. It is possible to fill the isthmus without being aware of it or to observe it microscopically filled with sealer or, less correctly, with sealer and dentinal debris (Figure 11). Fragments of separated endodontic instruments within root canals complicate or even prevent the removal of root canal filling materials. Attempts to unblock these root canals can lead to excessive force and perforations, as demonstrated in Figure 12. When two canals were very close and sometimes connected by isthmuses, a single photograph was taken to encompass both canals. Otherwise, separate photographs for each root canal were taken.

Tables 1 and 2 detail segments of root canals with unremoved filling materials and the presence of cracks or iatrogenic issues. Dentinal cracks were classified into reaching canal cracks (RCC) and non-reaching canal cracks (NRCC). This distinction is clinically significant because radial cracks that reach the root canal and sometimes the external surface of the root pose a high risk of vertical root fracture, a condition that usually necessitates extraction rather than conservative treatment.

As expected, the largest amount of unremoved root canal filling materials, intact root canal fillings, and iatrogenic issues were located in the apical third of the root canals. We concur with the view that magnification aids are essential not just for ensuring proper filling and removal of root canal materials but also for enhancing the understanding of the endodontic space. Recognizing intercanal isthmuses is significantly easier with the use of magnification.

In summary, this study underscores the complexities and challenges in removing root canal fillings, particularly in the apical third and in cases with secondary dentin deposition. The findings emphasize the need for thorough training and improved techniques to enhance the efficacy of endodontic retreatments.

### Future directions

Future research should aim to enhance training protocols for beginner operators, emphasizing the importance of proper instrumentation and irrigation techniques. Investigating more effective methods and tools for removing root canal filling materials, especially in complex canal anatomies, is crucial. Additionally, further studies should explore the properties and management of secondary dentin to mitigate risks during endodontic retreatment. These efforts will contribute to improved outcomes in endodontic therapy and retreatment, ultimately enhancing patient care.

## CONCLUSION

This study highlights the challenges faced by beginner operators in effectively removing root canal-filling materials. Residues of sealer and fragments of gutta-percha were frequently left behind, especially in cases where lateral condensation was used. The apical thirds of the root canals proved particularly problematic, reflecting the difficulties inherent in treating complex endodontic systems. The filling and removal processes in root canal isthmuses were identified as significant challenges. Key findings suggest that improper instrumentation, irrigation, or removal techniques contribute to the retention of materials on root canal walls and within sealers. The presence of secondary dentin, which can partially obstruct root canals, poses additional risks, including the potential for perforations during retreatment. The observed radial cracks likely result from premature use of endodontic files during initial canal shaping rather than during the removal of filling materials, despite the specific training provided to avoid excessive pressure.
